# Genetic and epigenetic variations associated with adaptation to heterogeneous habitat conditions in a deciduous shrub

**DOI:** 10.1002/ece3.3868

**Published:** 2018-02-05

**Authors:** Liu Lele, Du Ning, Pei Cuiping, Guo Xiao, Guo Weihua

**Affiliations:** ^1^ Institute of Ecology and Biodiversity College of Life Sciences Shandong University Jinan China; ^2^ College of Landscape Architecture and Forestry Qingdao Agricultural University Qingdao China

**Keywords:** Chinese chastetree, DNA methylation, ecological epigenetics, MSAP, phenotypic plasticity

## Abstract

Environmentally induced phenotypic plasticity is thought to play an important role in the adaption of plant populations to heterogeneous habitat conditions, and yet the importance of epigenetic variation as a mechanism of adaptive plasticity in natural plant populations still merits further research. In this study, we investigated populations of *Vitex negundo* var. *heterophylla* (Chinese chastetree) from adjacent habitat types at seven sampling sites. Using several functional traits, we detected a significant differentiation between habitat types. With amplified fragment length polymorphisms (AFLP) and methylation‐sensitive AFLP (MSAP), we found relatively high levels of genetic and epigenetic diversity but very low genetic and epigenetic differences between habitats within sites. Bayesian clustering showed a remarkable habitat‐related differentiation and more genetic loci associated with the habitat type than epigenetic, suggesting that the adaptation to the habitat is genetically based. However, we did not find any significant correlation between genetic or epigenetic variation and habitat using simple and partial Mantel tests. Moreover, we found no correlation between genetic and ecologically relevant phenotypic variation and a significant correlation between epigenetic and phenotypic variation. Although we did not find any direct relationship between epigenetic variation and habitat environment, our findings suggest that epigenetic variation may complement genetic variation as a source of functional phenotypic diversity associated with adaptation to the heterogeneous habitat in natural plant populations.

## 1 INTRODUCTION

Adaptation of plants to the heterogeneous environment may be achieved through natural selection of fixed traits and plasticity of variable traits (Pigliucci, 2001; Roda et al., 2013; Williams, 2008). Various studies have demonstrated the important role of ecologically relevant phenotypic plasticity in persistence across a range of habitats with spatial heterogeneity and temporal dynamics (Douhovnikoff & Hazelton, 2014; McLean et al., 2014; Nicotra et al., 2010). Although whether intraspecific variation in functional traits, most of which is assumed to be highly plastic, is more due to genetic control or epigenetic regulation remains essentially unexplored to date, emerging evidence has suggested that epigenetic mechanisms could facilitate phenotypic plasticity in response to ecologically relevant stressors under complex habitat conditions (Herrera & Bazaga, 2013; Medrano, Herrera, & Bazaga, 2014; Nicotra et al., 2015; Wilschut, Oplaat, Snoek, Kirschner, & Verhoeven, 2016; Zhang, Fischer, Colot, & Bossdorf, 2013).

Although there are several epigenetic mechanisms, including chemical modification of DNA and histones, position effects and interference by small noncoding RNAs (Richards, 2011), DNA methylation of cytosine is most the extensively studied epigenetic mechanism with important effects on ecologically relevant traits (Herrera & Bazaga, 2008, 2013; Schrey et al., 2013; Wilschut et al., 2016; Xie et al., 2015). While DNA is predominantly methylated at CG sites, cytosine methylations in plants occurs throughout the genome in all sequence contexts (CG, CHG and CHH where H = A, C or T) (Law & Jacobsen, 2010) and could affect whether transposons are silenced and genes are expressed. Biologists have illuminated that the amount and pattern of DNA methylation in model plants is sensitive to various environmental stressors under laboratory conditions, and ecologists have focused on the variation in DNA methylation in wild populations to understand the role of DNA methylation in plant adaptation to real environmental stress in nature (Bossdorf, Richards, & Pigliucci, 2008; Kilvitis et al., 2014). The rapidly increasing number of publications has illustrated that variation in DNA methylation is correlated with herbivory in violets (Herrera & Bazaga, 2010), salinity in marsh perennials (Foust et al., 2016), artificial disturbance in *Lavandula latifolia* (Herrera & Bazaga, 2016), metals in red maple (Kim, Im, & Nkongolo, 2016), and climate in *Quercus lobata* (Gugger, Fitz‐Gibbon, PellEgrini, & Sork, 2016; Platt, Gugger, Pellegrini, & Sork, 2015).

There is a complex relationship between genetic and epigenetic variation in the wild. Epigenetic variants can be under genetic control (Slotkin & Martienssen, 2007), but environmental factors can also directly alter the epigenetic variation that may be inherited through meiosis over several generations (Jablonka & Raz, 2009). The pattern of epigenetic variation in wild populations contributing to phenotypic traits which cannot be explained by genetic variation may be a consequence of natural selection on pre‐existing epigenetic variation, environmental induction of variable epigenetic variation, or both (Bossdorf et al., 2008; Verhoeven, vonHoldt, & Sork, 2016).

**Figure 1:**
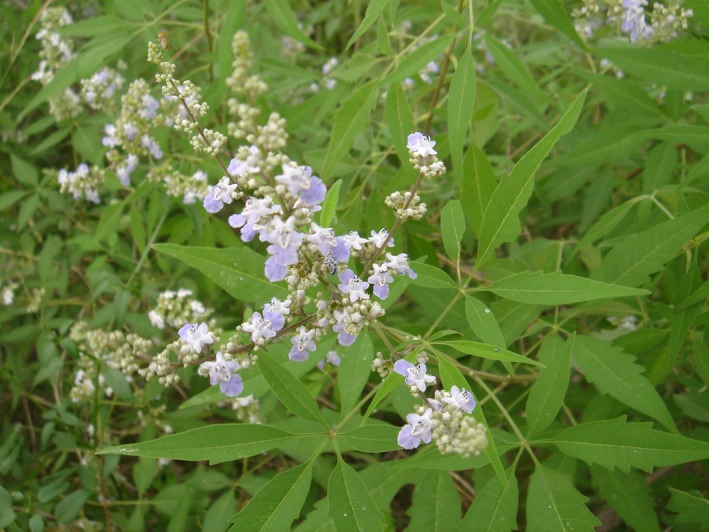
The study organism *Vitex negundo* var. *heterophylla*, a deciduous shrub with entomophilous flowers and digitate leaves containing five lanceolate leaflets, sometimes three

*Vitex negundo* var. *heterophylla* (Chinese chastetree, or five‐leaved chaste tree), a native deciduous officinal shrub with a heterophylly leaf and entomophilous flower (Figure 1), is widely distributed in the hilly areas of North China (Hu et al., 2015). *Vitex negundo* var. *heterophylla* is a typical species that can survive in a broad range of habitats, including bush and understory, varying in an array of other biotic and abiotic parameters (Li, Yang, & Wu, 2008). Chinese chastetree in roadside and bush habitats is frequently cut by farmers as firewood or to prevent it from occupying farmlands. It can adapt to cardinal environmental factors through modification of a series of morphological and physiological characteristics (Du, Guo, Zhang, & Wang, 2010; Du et al., 2012). As a long long‐lived perennial, *V. negundo* var. *heterophylla* may face variable environments through epigenetic processes (Bräutigam et al., 2013). Therefore, it provides an ideal study system to compare genetic and epigenetic differences in response to heterogeneous habitat conditions.

In this study, we measured several plant functional traits for individuals sampled from each plot to determine habitat differentiation in phenotype. We investigated the genetic variation using amplified fragment length polymorphism (AFLP) and epigenetic variation using methylation‐sensitive AFLP (MSAP). These dominant markers provided a considerable number of anonymous loci, which generate powerful data for detecting the genetic and epigenetic structure across several heterogeneous habitats in a nonmodel species without a reference genome. We compared the patterns of genetic and epigenetic variation shaped by the habitat conditions to test the hypothesis that epigenetic variation plays a potential role independent of genetic variation in the adaptation of *V. negundo* var. *heterophylla* to the environment.

## 2 MATERIALS AND METHODS

### 2.1 Sampling design

**Figure 2:**
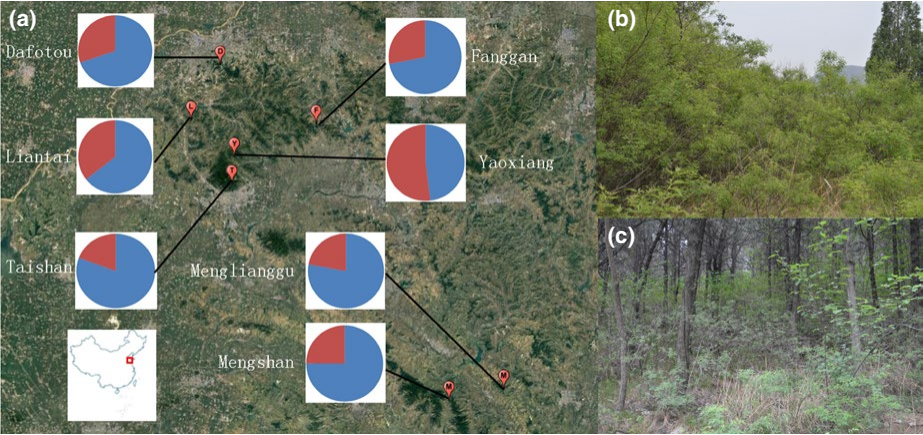
(a) Maps of the seven *Vitex negundo* var. *heterophylla* sampling sites in Shandong Province, China, with the results of Bayesian clustering from STRUCTURE. The shaded portion of the circle indicates population assignment to two groups. Examples of (b) the open habitat and (c) the forest understory habitat

We selected plots for this study from seven sites in the south hills of Shandong Province, eastern China, separated by approximately 20–150 km (Figure 2). For each site, we collected samples from two adjacent plots with contrasting habitat conditions at a distance of <2 km (Table 1) in May 2016. At each plot, 13–15 widely spaced individuals were randomly chosen and marked with permanent tags. To avoid developmental epigenetic variation confusing the differences among heterogeneous environments, all leaf samples for molecular analyses were picked at the same position of the plant before the flowering phase. Young expanding leaves were collected from each plant, placed in paper envelopes, and dried immediately with silica gel. This material was used for genomic DNA extraction, and additional expanding leaves were randomly collected to measure the leaf traits.

**Table 1:** Overview of sampled populations of *Vitex negundo* var. *heterophylla*

Population	Sample size	Site	Latitude (°N)	Longitude (°E)	Habitat Type	Dominant tree
D1	14	Dafotou	36.63179	117.0347	Understory	*Platycladus orientalis*
D2	14	Dafotou	36.62825	117.0507	Open	
F1	15	Fanggan	36.43172	117.4516	Understory	*Quercus acutissima, Robinia pseudoacacia*
F2	14	Fanggan	36.43333	117.4515	Open	
L1	14	Liantai	36.44292	116.937	Understory	*Cotinus coggygri, Robinia pseudoacacia, Quercus acutissima*
L2	13	Liantai	36.44398	116.9425	Open	
Y1	14	Yaoxiang	36.32133	117.12	Understory	*Quercus acutissima*
Y2	14	Yaoxiang	36.31391	117.1388	Open	
M1	14	Mengshan	35.5376	117.9895	Understory	*Pinus armandii, Robinia pseudoacacia*
M2	14	Mengshan	35.52588	117.987	Open	
N1	13	Menglianggu	35.57246	118.2078	Understory	*Pinus armandii*
N2	14	Menglianggu	35.57203	118.2214	Open	
T1	14	Taishan	36.2313	117.1125	Understory	*Quercus acutissima, Robinia pseudoacacia, Platycladus orientalis*
T2	14	Taishan	36.2257	117.1141	Open	

### 2.2 Measurement of plant functional traits

For each sampled individual, plant maximum height (H), basal diameter (D), and number of resprouts (NR) were measured, and more than five fully expanded compound leaves were collected at different positions of the stem. Both leaf fresh weight (LFW) and leaf dry weight (LDW) were measured and leaf water content [LWC = (LFW − LDW)/LDW] was obtained. Leaflet area (Area) was determined with image processing program ImageJ (Abràmoff, Magalhães, & Ram, 2004), and the specific leaf area (SLA = LA/LDW) was calculated. The number of resprouts (NR) was divided into three levels to assess the environmental disturbance (Pérez‐Harguindeguy et al., 2013): level 0 for one stem, level 1 for more than one but <5 stems, and level 2 for more than five stems. Finally, the coefficient of height‐diameter allometry (HDA) was calculated for each population using function MSA(D~H, log = “xy”) in R package smart (Warton, Duursma, Falster, & Taskinen, 2012).

### 2.3 AFLP and MS‐AFLP protocol

We investigated a total of 195 individuals for genetic and epigenetic variation with AFLP and MSAP using the same DNA sample for each individual. Total genomic DNA was extracted from dried leaf tissue according to the cetyl trimethylammonium bromide (CTAB) method (Doyle & Doyle, 1987) with some modifications. PVP 40,000 was used to improve DNA yield and quality. DNA was quantified with both 0.8% agarose gels and microscopic spectrophotometry.

The protocol for MSAP was adapted from a standard AFLP (Vos et al. 1995), replacing the *Mse*I enzyme in two separate runs with the methylation‐sensitive enzymes *Hpa*II and *Msp*I using appropriate adaptors and primers. For restriction digests, 2 μl genomic DNA (ca. 150 ng) was combined with 10 μl double digestion mix containing 1 μl 10 × CutSmart Buffer (New England Biolabs, NEB), 2.5 μ*Eco*RI (NEB), 0.5 μ*Mse*I or 2.5 μ*Hpa*II or 2.5 μ*Msp*I (NEB) in parallel reactions. The reaction was incubated at 37°C for 2 hr and inactivated at 80°C for 20 min. Then, the product was combined with 48 μT4 DNA ligase (NEB), 1.4 μl 10 × T4 DNA ligase buffer, 1 μl *EcoR*I adapter (5 μM), and 1 μl *Mse*I adapter (50 μM) or 1 μl *Hpa*II*/Msp*I adapter (50 μM). The reaction was incubated at 16°C for 2 hr, inactivated at 65°C for 10 min, and diluted 1:5. For the preselective amplification (PCR1), 2 μl ligation product was combined with 13 μl PCR1 reaction mix containing 0.7 μl preselective primers (5 μM) each, 0.6 μl dNTPs (TIANGEN, 2.5 mM each), 1.5 μl 10× buffer (TIAGEN), 0.75 U polymerase (TIAGEN), and 9.2 μl H_2_O. The thermocycler protocol was 72.0°C (2 min) followed by 20 cycles of 94.0°C (20 s), 56.0°C (30 s), and 72.0°C (2 min) and a final extension at 60.0°C (30 min), performed on a Biometra TGradient. The PCR1 product was diluted 1:5. For the selective amplification (PCR2), 2 μl PCR1 product was combined with 13 μl PCR2 reaction mix containing 0.7 μl selective primers (5 μM) each, 0.6 μl dNTPs (TIANGEN, 2.4 mM each), 1.5 μl 10 × buffer (TIAGEN), 0.75 U polymerase (TIAGEN), and 9.2 μl H_2_O. The thermocycler protocol was 94.0°C (2 min) followed by 10 cycles of 94.0°C (20 s), 66.0°C (30 s, decreasing 1°C per cycle) and 72.0°C (2 min) and 20 cycles of 94.0°C (20 s), 56.0°C (30 s) and 72.0°C (2 min), and a final extension at 60.0°C (30 min). Six selective primer combinations were chosen for the AFLP (*Eco*RI + ACA and *Mse*I + CTA, *Eco*RI + ACT and *Mse*I + CAT, *Eco*RI + ACC and *Mse*I + CTC) and MSAP (*Eco*RI + AAC and *Hpa*II/*Msp*I + TC, *Eco*RI + ACG and *Hpa*II/MspI + TC, *Eco*RI + ACT and *Hpa*II/*Msp*I + TA) analyses (Table 2), of which the *Eco*RI + 3 primers were 5′‐end labeled with FAM dye.

**Table 2:** Adapter and primer sequences for AFLP and MSAP amplification

Primer	Sequence(from 5'to 3’)
Adapters	
*Eco*RI_adapter top	CTCGTAGACTGCGTACC
*Eco*RI_adapter bottom	AATTGGTACGCAGTCTAC
*Mse*I_adapter top	GAGCGATGAGTCCTGAG
*Mse*I_adapter bottom	TACTCAGGACTCAT
*Hpa*II/*Msp*I_adapter top	GATCATGAGTCCTGCT
*Hpa*II/*Msp*I_adapter bottom	CGAGCAGGACTCATGA
Preselective primers	
*Eco*RI+A	GACTGCGTACCAATTCA
*Mse*I+C	GATGAGTCCTGAGTAAC
*Hpa*II/*Msp*I	ATCATGAGTCCTGCTCGG
Selective primers	
*Eco*RI+AAC^4^	GACTGCGTACCAATTCAAC
*Eco*RI+ACA^1^	GACTGCGTACCAATTCACA
*Eco*RI+ACT^2,6^	GACTGCGTACCAATTCACT
*Eco*RI+ACC^3^	GACTGCGTACCAATTCACC
*Eco*RI+ACG^5^	GACTGCGTACCAATTCACG
*Mse*I+CAT^2^	GATGAGTCCTGAGTAACAT
*Mse*I+CTA^1^	GATGAGTCCTGAGTAACTA
*Mse*I+CTC^3^	GATGAGTCCTGAGTAACTC
*Hpa*II/*Msp*I+TA^6^	ATCATGAGTCCTGCTCGGTA
*Hpa*II/*Msp*I+TC^4,5^	ATCATGAGTCCTGCTCGGTC

Superscript numbers indicate primer combinations used for the selective amplification, and every primer combination is tagged with same number.

The final selective PCR productions were separated and visualized on an ABI3730XL DNA capillary sequencer (Applied Biosystems, Foster City, USA) with Rox‐500 internal size standard (Applied Biosystems) at the Shandong Academy of Agricultural Sciences. We used PEAKSCANNER v1.0 (Applied Biosystems) to analyze the AFLP and MSAP profiles. Binning of fragments was performed using a peak height threshold of 50 relative fluorescence units and a minimal size of 50 base pairs. Peak height data were scored with a binary code, zero for band absent, and one for band present. For every polymorphic locus, each allele must exist in more than two individuals (>1% of all samples).

### 2.4 Data analysis

To identify how many different genetic groups are represented in our collection regardless of the geographic sampling location, we performed Bayesian clustering of genetic data using STRUCTURE v2.3.4 (Falush, Stephens, & Pritchard, 2007; Pritchard, Stephens, & Donnelly, 2000). Structure estimates the number of groups (*K*) present among individuals and assigns individuals to each *K* using Bayesian modeling. We tested 20 populations (*k* = 1–20), which are more than the maximum anticipated based on sampling location, with 20 runs at each *k*. We used both the log probability of observing the data (Ln Pr(*x*|*k*)) method of Structure and Delta *K* (Evanno, Regnaut, & Goudet, 2005) with the online program STRUCTURE HARVESTER for visualizing STRUCTURE output and implementing (Earl & vonHoldt, 2012), which determines the number of populations that best fit the data. We incorporated sampling locations in our model to assist in detecting sampling weak differentiation. We performed clustering with the admixture model, 30,000 burn‐in steps, 100,000 postburn‐in steps, and allowed correlated allele frequencies. We assigned individuals to groups based on the highest *q*‐value.

All basic statistical analyses were carried out using the R environment. The MSAP profiles were analyzed with the R script MSAP_calc (Schulz, Eckstein, & Durka, 2013) using the function Extract_MSAP_epigenotypes with parameters Epicode = “Mix1,” delete.monomorphic.loci = TRUE, and MinPoly = 2. Under the scoring scheme “Mix1,” M‐MSAP makers were obtained with a methylation scoring approach scoring the presence of both *Eco*RI–*Hpa*II and *Eco*RI–*Msp*I products as “1” and scoring other conditions as “0,” while u‐MSAP makers were transformed with a nonmethylation scoring approach treating the presence of only the EcoRI–MspI fragment (hemi‐ or fully methylated at the internal cytosine) or only the EcoRI–MspI fragment (hemi‐methylated at the external cytosine) as “1” and treating other conditions as “0.”

To estimate the amount of epigenetic and genetic variation, Shannon's diversity index (H) and percentage of polymorphisms (PPL) for each population were calculated with GENALEX 6.5 (Peakall & Smouse, 2006). We also used GENALEX to estimate genetic and epigenetic differentiation using hierarchical analysis of molecular variation (AMOVA) to determine whether spatial location structured genetic or epigenetic differences by comparing the genetic and epigenetic variation among sites (ФRT), among populations (i.e., habitats) within sites (ФPR), and within populations (ФPT). We used 9,999 permutations to estimate statistical significance and an initial alpha of 0.05. Moreover, we used generalized linear models (GLM) for each loci to determine the specific AFLP and MSAP loci correlated with the habitat type using function glm in R.

We analyzed the correlation between genetic variation, epigenetic variation, and habitat by performing Mantel and partial Mantel tests using zt software (Bonnet & Van de Peer, 2002). Using a simple Mantel, we compared the genetic distance matrix to the epigenetic distance matrix to test for a relationship between genetic and epigenetic variation. While a Mantel test determines correlations between two distance matrices, the partial Mantel test determines correlations between two distance matrices while controlling for correlations with a third matrix. In this case, we used a partial Mantel to test for an independent relationship between genetic variation and habitat while controlling for epigenetic variation. Likewise, we tested for a relationship between epigenetic variation and habitat while controlling for correlations with genetic variation. To create the habitat distance matrix, two different strategies were adopted. For the first habitat distance matrix, we used zero to indicate understory habitat and one to indicate open habitat. The second one was based on the fitness‐related traits, including SLA, LWC, NR, and HDA, performed using function dist (method = “euclidean”) in R software. Both strategies make the assumption that differences between habitats will be essentially the same magnitude regardless of individual population differences. In all cases, we used the Euclidean genetic and epigenetic distance matrices generated by GENALEX. We also constructed Euclidian geographic distances and Nei unbiased genetic and epigenetic distances to test the roles of major role for population differentiation. As simple and partial Mantel tests have been questioned for a number of drawbacks (Bradburd, Ralph, & Coop, 2013; Guillot & Rousset, 2013; Legendre, Fortin, & Borcard, 2015), we applied multiple matrix regression with randomization (MMRR) (Wang, 2013) as an alternative approach to Mantel procedures. Computations were implemented in with the MMRR function using 9,999 permutations.

## 3 RESULTS

### 3.1 Differences in plant functional traits

**Figure 3:**
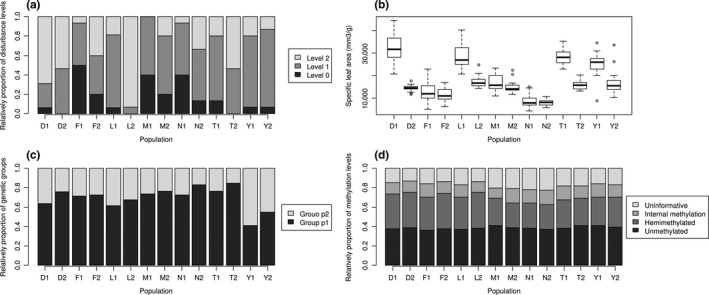
Differences among population in disturbance (a), specific leaf area (b), genetic structure (c), and DNA methylation (d). (a) The individual with only one resprout indicates a minor disturbance (level 0), the individual with 2–5 resprouts indicates a medium disturbance (level 1), and the individual with more than five resprouts indicates a recent severe disturbance (level 2). (b) Specific leaf area is significantly different between habitats in most sites and varies among sites. (c) The genetic assignment of group p1 in open habitat is slightly higher than that in understory habitat within every site. (d) There is no significant difference in DNA methylation level between habitats or among sites

**Figure 4:**
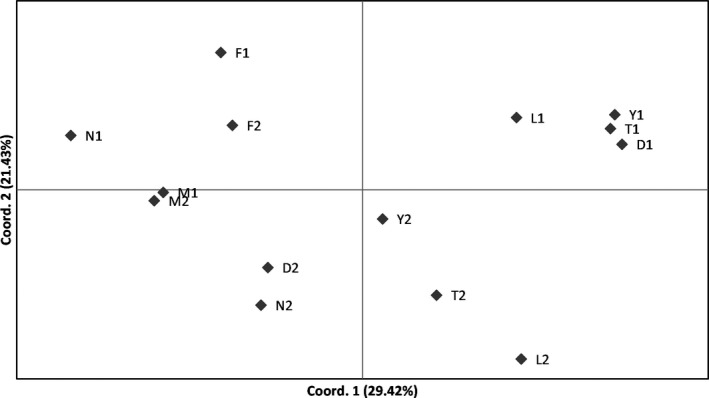
Principal coordinates analyses (PCoA) of distances for functional traits of 14 populations of *Vitex negundo* var. *heterophylla*

At the population level, we found significant variation for D [paired *t*‐test: *t* (6) = −2.51, *p* = .046], LWC [paired *t*‐test: *t*(6) = 3.19, *p* = .019] and SLA [paired *t*‐ test: *t*(6) = 2.96, *p* = .025], and similar patterns of disturbance between two populations were revealed among sites other than Dafotou and Yaoxiang (Figure 3a). At the individual level, significant differences between two habitats were detected for LWC and SLA (Figure 3b) at four sites: Dafotou, Yaoxiang, Liantai, and Taishan (Table 3). Principal coordinates analyses (PCoA) displayed different phenotypic divergences among sites, indicating complex heterogeneous habitats. The cord. 2 in PCoA may suggest the phenotypic variation between two habitat types (Figure 4).

**Table 3:** T‐value for individuals between two populations for each site

	Dafotou	Fanggan	Yaoxiang	Liantai	Mengshan	Menglianggu	Taishan
Height	−1.732	1.531	−0.806	−1.625	1.638	0.231	−1.852
Diameter	−3.187	−0.702	−1.663	−3.218*	−0.310	0.087	−0.4030*
LWC	3.390*	2.014	2.903*	3.651**	−0.736	3.967**	3.703**
SLA	10.13**	0.658	3.604*	6.778**	1.278	0.655	11.85**

*p < .01; **p < .001.

### 3.2 Genetic diversity and structure

The AFLP analysis resulted in 142 polymorphic loci across which every individual displayed a unique genotype. Values of diversity and percent polymorphisms across populations are given in Table 4. These loci identified that genetic diversity across all populations was high (h‐AFLP ranged from 0.408 to 0.498). There was no difference in genetic diversity between understory and open habitats (paired *t*‐test: *t* = 0.60723, *df* = 6, *p*‐value = .566 for H; *t* = 0.85722, *df* = 6, *p*‐value = .4242 for PPL). We detected population structure at every level of hierarchy and found significant variation among regions (explaining 7% of the genetic variance) and among populations within region (2%), and the most (91%) among individuals within population (Table 5).

**Table 4:** Values of diversity (h) and percent polymorphisms (%P) of populations

Pop	Sample size	h‐AFLP	%P‐AFLP	h‐u‐MSAP	%P‐u‐MSAP	h‐m‐MSAP	%P‐m‐MSAP
D1	14	0.54	84.51	0.471	95.89	0.563	100.00
D2	14	0.463	73.24	0.477	97.26	0.562	99.31
F1	15	0.496	78.17	0.449	95.89	0.566	100.00
F2	14	0.537	85.21	0.467	95.89	0.566	99.31
L1	14	0.436	71.83	0.460	96.58	0.551	100.00
L2	13	0.495	78.87	0.471	95.21	0.545	98.61
M1	14	0.506	81.69	0.485	94.52	0.495	100.00
M2	14	0.501	80.28	0.453	92.47	0.509	98.61
N1	14	0.487	78.87	0.469	95.89	0.503	98.61
N2	14	0.459	73.24	0.447	94.52	0.502	98.61
T1	13	0.462	73.94	0.463	92.47	0.538	99.31
T2	14	0.408	65.49	0.481	94.52	0.515	98.61
Y1	14	0.600	97.18	0.493	100.00	0.535	99.31
Y2	14	0.586	93.66	0.476	95.89	0.531	100.00
Mean	13.929	0.498	79.73	0.469	95.50	0.534	99.31
SE	0.010	0.006	2.28	0.004	0.51	0.003	0.16

**Table 5:** Three‐level hierarchical analysis of molecular variance (AMOVA) for AFLP, u‐MSAP and m‐MSAP data sets among sites, among populations within sites (among Pops,) and within populations (within Pops)

Source	*df*	% variation	Ф‐statistics
Genetic variation based on AFLP			
Among sites	6	7	0.067**
Among Pops	7	2	0.086**
Within Pops	181	91	0.020*
Epigenetic variations based on u‐MSAP			
Among Sites	6	2	0.020**
Among Pops	7	1	0.011*
Within Pops	181	97	0.030**
Epigenetic variations based on m‐MSAP			
Among Sites	6	2	0.023**
Among Pops	7	0	0.0005
Within Pops	181	98	0.024**

*df*, degrees of freedom.

Ф‐statistics were calculated using 9,999 permutations.

*p ≤* *.01, **p ≤ .0001.

Bayesian clustering identified only two genetic groups (Delta *K* = 184.07), which indicated a high degree of intermixing between populations. These groups did not clearly reflect geographically based differences among seven sites (Figure 2a), but significant differentiation between understory and open habitats was detected in the amount of each of the two groups [paired *t*‐test: *t*(6) = −4.39, *p* = .005] (Figure 3c). We found 15 AFLP loci correlated with habitat type in replicate populations using GLM.

### 3.3 Epigenetic diversity and structure

A total of 146 MSAP loci were analyzed, which were transformed into 146 u‐MSAP loci and 146 M‐MSAP. All 195 individuals displayed unique u‐MSAP and M‐MSAP genotypes. The epigenetic diversity was high based on both u‐MSAP (ranged from 0.449 to 0.493) and m‐MSAP (ranged from 0.495 to 0.566). There was also no significant difference in epigenetic diversity between understory and open habitats. We found higher levels of epigenetic than genetic diversity at the population level using index PPL [*t*(13) = −8.799, *p* < .001 for m‐MSAP; *t*(13) = −7.591, *p* < .001 for u‐MSAP], but two MSAP scoring approaches drew different conclusions comparing index H. Genetic H was lower than epigenetic H calculated with m‐MSAP data [paired *t*‐test: *t*(13) = −2.423, *p* = .031], but higher with u‐MSAP data [paired *t*‐test: *t*(13) = 2.193, *p* = .047]. However, we failed to detect a significant correlation between genetic and epigenetic diversity. For u‐MSAP, hierarchical AMOVA detected little variation (1%) among populations within a site, but failed for m‐MSAP, and most variation existed within population. Moreover, MSAP datasets did not infer significant differences in genome‐wide cytosine methylation level (Figure 3d) between heterogeneous habitats [paired *t*‐test: *t*(6) = −0.2443, *p* = .8151]. Only one MSAP locus was determined with habitat type through analysis with GLM.

### 3.4 Comparison between genetic and epigenetic variation

**Figure 5:**
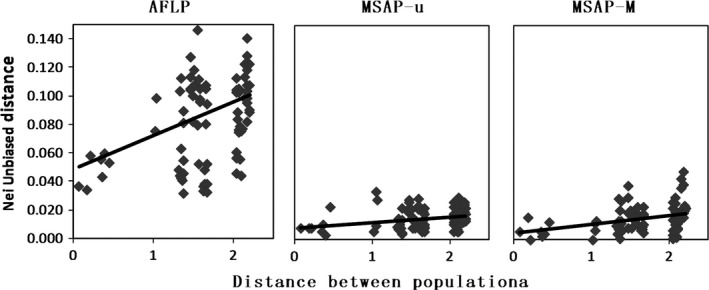
Fitted linear regressions for the genetic (AFLP) and epigenetic (u‐MSAP and m‐MSAP) markers, depicting the relationship between pairwise Nei unbiased distance and spatial separation (note logarithmic scale) for the *N* = 14 populations

Simple Mantel tests showed significant correlations between genetic and epigenetic variation across all populations (Table 6). Both simple Mantel tests (Table 6) and MMRR (Table 7) showed that phenotypic variation was correlated with epigenetic variation, but not with genetic variation. Simple Mantel tests found no relationship between habitat type and genetic or epigenetic variation, but MMRR showed a possible association between genetic differentiation and habitat types (*r* = .081, *p* = .0485). We also detected the geographic structure of genetic and epigenetic variation. At the level of population, we found a lower geographic regression slope for epigenetic structure than genetic structure using the Mantel method (β_AFLP_ = 0.0234, β_MSAP‐u_ = 0.0042, β_MSAP‐m_ = 0.0062; Figure 5). The above results obtained from u‐MSAP and m‐MSAP markers separately showed the same trends (Table 6). MMRR also revealed the analogous genetic and epigenetic spatial structure (Table 8).

**Table 6:** Correlation coefficients using simple Mantel tests across all sites

	Gen	Epi_u	Epi_m
Epi_u	0.110*	–	–
Epi_m	0.055*	0.344**	–
Env	−0.004	0.0005	−0.012
Phe	0.044	0.046*	0.071**
Geo	0.063**	0.051**	0.086**

Gen, genetic variation; Epi_u, epigenetic variation using u‐MSAP; Epi_m, epigenetic variation using m‐MSAP; Env, environment; Phe, phenotype; Geo, geographical distance.

*p ≤ .05; **p ≤ .001.

**Table 7:** Summary of multiple matrix regression analysis with randomization (MMRR) relating the phenotypic distance matrix with genetic and epigenetic distance matrices

Differentiation matrix	Epigenetic marker used	Overall regression		Linear predictor matrices			
				Genetic distance		Epigenetic distance	
		*F*	*p*	Coefficient	*p*	Coefficient	*p*
Phenotype	u‐AFLP	35.91	.0484	0.0032	.1537	0.0057	.0525
Phenotype	m‐AFLP	63.81	.005	0.0034	.1484	0.0118	.0001

*p* values were calculated with 9,999 permutations.

**Table 8:** Summary of multiple matrix regression analyses with randomization (MMRR) relating genetic, epigenetic, and phenotypic distance matrices with geographical and environmental distance matrices

Differentiation matrix	Overall regression		Linear predictor matrices			
			Geographical distance		Environmental distance	
	*F*	*p*	Coefficient	*p*	Coefficient	*p*
Gen	37.91	.0009	1.300	.0008	0.181	.0485
Epi‐u	25.38	.0011	0.650	.0011	−0.185	.7992
Epi‐m	70.44	.0001	0.843	.0001	−0.940	.3175
Phe	8055	.0001	109.0	.0001	61.18	.0001

Gen, genetic variation using AFLP markers; Epi_u, epigenetic variation using u‐MSAP; Epi_m, epigenetic variation using m‐MSAP; Phe, phenotype.

*p* values were calculated with 9,999 permutations.

**Figure 6:**
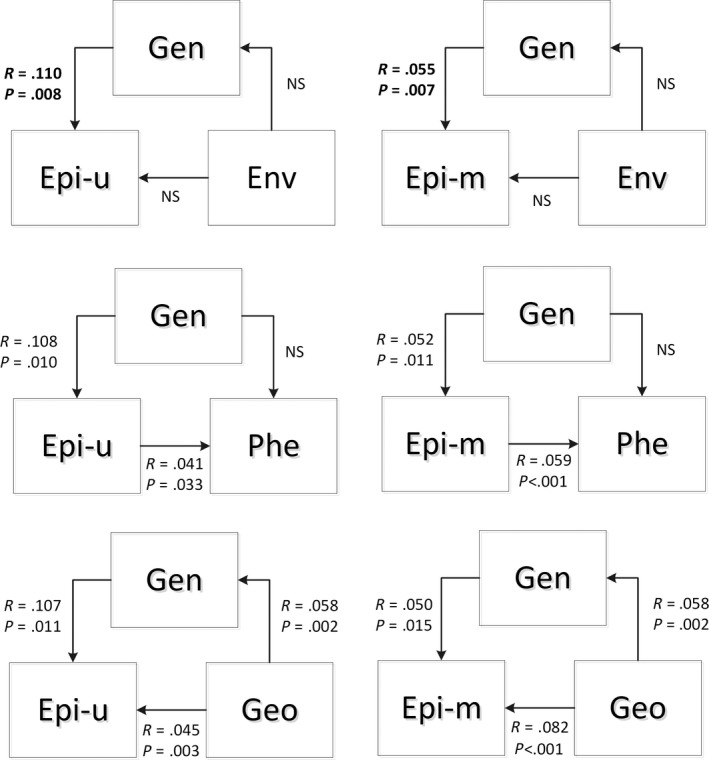
Genetic and epigenetic correlations to variation in habitat environment, functional phenotype, and geographic distance using partial Mantel tests with the Euclidean genetic and epigenetic distance matrices, habitat distance matrices, and trait distance and geographical distance matrices for all individuals across all populations. The correlations between genetic and epigenetic variation were calculated in separate partial Mantel tests. Gen = genetic variation, Epi‐u = Epigenetic variation based on MSAP‐u, Epi‐m = Epigenetic variation based on MSAP‐M, Env = environmental variation, Phe = phenotypic variation, Geo = geographical distance, NS = not significant, *r* = correlation coefficient when significant and *p*‐value

Partial Mantel tests always displayed a significant correlation (*r* > .10, *p* < .05 for u‐MSAP; *r* ≥ .05, *p* < .05 for m‐MSAP) between genetic and epigenetic variation when we controlled for the additional factor (Figure 6). However, we detected no relationship between habitat type and genetic variation when we controlled for epigenetic variation and no relationship between habitat type and genetic variation when we controlled for epigenetic variation. Moreover, we did not find a significant correlation between phenotypic and genetic variation when we controlled for epigenetic variation, but we found a significant correlation (*r* = .041, *p* = .033 for u‐MSAP; *r* = .059, *p* < .001 for m‐MSAP) between phenotypic and epigenetic variation when we controlled for genetic variation. Additionally, we detected the spatial structure of genetic (*r* = .058, *p* = .002 with controlled u‐MSAP; *r* = .058, *p* = .005 with controlled m‐MSAP) and epigenetic (*r* = .045, *p* = .003 for u‐MSAP; *r* = .082, *p* < .001 for m‐MSAP) diversity.

## 4 DISCUSSION

Consistent with the previous studies of *V. negundo* (Su et al., 2003; Zhang, Zheng, & Ge, 2007), we found high levels of genetic diversity and low population genetic differentiation both among and within sites. In particular, Bayesian clustering revealed a significant genetic differentiation shaped by habitat heterogeneity. We also found high levels of epigenetic diversity but little epigenetic structure among populations within site, and random levels of DNA methylation across all populations. This result is consistent with the previous findings in salt marsh perennials (Foust et al., 2016), suggesting that genetic variation may be more strongly structured by environment than epigenetic variation. Although the scoring approaches are largely consistent when comparing the levels of diversity among populations, estimates of epigenetic diversity varied strongly (Schulz et al., 2013), and the deviation made by scoring approaches has observably affected our comparison of levels between genetic and epigenetic diversity. Consistent with previous studies of other species distributed across different habitats observing equal or higher epigenetic than genetic diversity (Foust et al., 2016; Herrera & Bazaga, 2010; Richards, Schrey, & Pigliucci, 2012; Schulz, Eckstein, & Durka, 2014), we found slightly higher levels of epigenetic than genetic diversity in *V. negundo* var. *heterophylla*. Although little differentiation between habitats within sites indicated strong gene flow, the AMOVA, Bayesian, and GLM analyses concluded congruently that genetic variation may play a more important role in habitat differentiation than epigenetic variation. The study of a floodplain herb emphasized the major role of environmentally induced epigenetic variation for adjustment to changing habitat conditions (Schulz et al., 2014), but our results indicated a very marginal role of epigenetic variation in the perennial shrub.

The main distinction between understory and open habitat is the availability of light, which may have strong effects on a series of other biotic and abiotic environmental factors. In fact, there are many other obvious environmental differences in arbor coverage, human disturbance, elevation, slope aspect, and climate, which we cannot control for in natural conditions across seven sites. Moreover, the understory and open habitats are dynamic and could rapidly transform. Although epigenetic variation is considered as a very potential component in the adaption to the changing environment, relationships among genetic and epigenetic variation and habitat may show different patterns in varying species. When running simple and partial Mantel tests, we obtained the same pattern as for *Borrichia frutescens* (Foust et al., 2016), failing to detect any relationship between epigenetic and habitat environment using crude binary data to describe complex habitats, as we had a larger number and range of sampling plots than the previous studies (Foust et al., 2016; Kim et al., 2016; Robertson, Schrey, Shayter, Moss, & Richards, 2017; Schulz et al., 2014). Limited studies have been conducted of epigenetic differentiation in natural populations across heterogeneous habitat conditions, and there is an urgent need to develop or replace the binary method to comprehensively characterize the complex habitat conditions. Here, we tried to introduce the functional traits for representing the environment.

The selected traits, including leaf water content, specific leaf area, level of resprout, and height‐diameter allometry, were plastic and could respond to the heterogeneous environment. For example, SLA, an indicator of ecophysiological characteristics with a strong phenotypic plasticity and substantial genetic effects (Scheepens, Frei, & Stöcklin, 2010), would increase in the understory habitat to optimize light harvesting (McIntyre & Strauss, 2014), as supported by our data. In addition, NR could be considered as a typical parameter of branching architecture for the shrub, which differs on the basis of browsing history, fire history, and other types of disturbance (Pérez‐Harguindeguy et al., 2013), especially access to light, water stress, and the human‐caused cutting for *V. negundo* var. *heterophylla* in our research region. We found phenotypic plasticity in response to controlled drought and shade treatment in our previous glasshouse experiments using *V. negundo* var. *heterophylla* (Du et al., 2010, 2012), and this field experiment also displayed adaptive plasticity in response to heterogeneous wild habitats. Nevertheless, assessing plant functional trait data separately may misrepresent the adaptive response to the complex habitat. Thus, we combined several typical fitness‐related phenotypes to quantify the divergence of adaptions.

Amazingly, we found 15 AFLP loci statistically correlated with habitat type, and the Bayesian clustering suggested a parallel genetic divergence that may result in microevolution from independent origins. This was also supported by MMRR showing potential effect of habitat environment on genetic differentiation. Although strong gene flow has homogenized the genetic differentiation across the genome, adaptive loci will remain due to similar pressure (Trucchi, Frajman, Haverkamp, Schönswetter, & Paun, 2017). Only several plastic functional traits were investigated, and very limited loci were detected by AFLP. Therefore, to test this hypothesis, it is necessary to find the adaptive phenotypes and adopt next‐generation sequencing methods. We did expect epigenetic differentiation between open and understory populations, but we only found one locus showing differentiation due to habitat type. Moreover, it remains a question whether this locus plays an independent role from genetic variation in adaptation to diverse habitats. It is possible either that the understory environment did not change any epigenetic variation between habitats, or that any existing epigenetic signature was too weak to be detected given the high epigenetic diversity between individuals among all populations.

The epigenetic mechanism may be restricted when natural plant populations endure some discrete human‐caused disturbance, such as heavy metal pollution (Kim et al., 2016), experimental disturbance (Herrera & Bazaga, 2016), and oil spills (Robertson et al., 2017). According to our field investigations and planting experiments (Du et al., 2012), Chinese chastetree tends to distribute in open habitats with plentiful light. Understory individuals might sometimes undergo aboveground dieback due to shade and drought, but individuals in open habitat were disturbed by human‐caused cutting in five (Fanggan, Liantaishan, Mengshan, Menglianggu, and Taishan) of seven study sites (see Figure 3a). Unlike the cyclic pattern of environmental factors such as light, rainfall, and salinity, plants may have not experienced these artificial stressors in evolutionary history. If plants do not have a plastic or regulatory response such as the epigenetic mechanism, genetic diversity is the vital resource of adaptive phenotypes and the genetic structure will be shaped deeply.

It is a challenge to search for epigenetic components independent from genetic variation in *V. negundo* var. *heterophylla* with such high genetic diversity. While many previous studies took advantage of low genetic diversity clonal or inbred species to minimize possibilities for genetic control (Richards et al., 2012; Schulz et al., 2014; Verhoeven, Jansen, van Dijk, & Biere, 2010), some recent studies attempted to use statistical approaches to uncover patterns of epigenetic variation that are not predictable from patterns of genetic variation (Foust et al., 2016; Gugger et al., 2016; Herrera, Medrano, & Bazaga, 2016). Simple and partial Mantel tests were proposed to combine the analyses of genetic and epigenetic variation and present the correlation between genetic or epigenetic variation and habitat while controlling for the correlation between genetic and epigenetic variation (Foust et al., 2016). A controlled planting experiment investigating epigenetic variation in response to warming failed to find significant correlations between epigenetic variation and phenotype or habitat using Mantel tests (Nicotra et al., 2015). In contrast, we found a weak but significant correlation between epigenetic variation and adaptive phenotype, with no significant correlation between genetic and adaptive phenotypes. The results indicated that epigenetic mechanisms may play a more important role in adaptive plasticity than genetic variation in some scenarios.

Finding direct casual links between epigenetic and variation and ecological phenotype is a key challenge in the study of epigenetic adaptation with nonmodel species (Richards et al., 2017). A glasshouse experiment with various epigenetic recombinant inbred lines of *Arabidopsis thaliana* concluded that heritable epigenetic variation can cause substantial variation in ecologically important plant traits (Zhang et al., 2013). To investigate the relationship between epigenetic variation and functional plant diversity in wild populations, genetic and epigenetic marker–trait association analyses for 20 functional traits in a perennial herb were conducted, showing that more MSAP markers than AFLP involved in significant association (Medrano et al., 2014). Due to the limited loci and phenotypes in our study, we did not perform a genotype–phenotype association analysis. Bayesian clustering and GLM revealed the genetic differentiation between habitats, but Mantel tests and MMRR indicated a significant correlation between epigenetic and phenotypic variation. This contradictory result suggests that the key stable phenotypes associated with habitat type were not considered in our study, as we only measured several plastic functional traits varying not only between habitats but also among all sites. However, our results added the indirect evidence that epigenetic variation can serve as an important source of intraspecific functional diversity in nature.

According to the illustration of genetic and epigenetic isolation‐by‐distance scenarios along with hypothesized causes (Herrera et al., 2016), our data revealed the moderate recent epigenetic variation between generations in natural populations of *V. negundo* var. *heterophylla*. Consistent with the results from a perennial herb (Herrera, Medrano, & Bazaga, 2017), geographic distance explained genetic differentiation better than epigenetic differentiation, and epigenetic variation contributed to the divergence in functional traits in the perennial shrub. It is necessary to conduct transplantation or common garden experiments with offspring from populations in different habitats to distinguish the contribution of induced and inherited epigenetic variation to adaptive phenotypes. As the MSAP marker only provides limited anonymous loci that are difficult to link to functional genomic elements or phenotype, a reduced representation bisulfite sequencing approach based on next‐generation sequencing methods is the next level of epigenetic study in natural populations (Gugger et al., 2016; Platt et al., 2015; Robertson & Richards, 2015; Trucchi et al., 2016).

## 5 CONCLUSION

Our study used functional traits, AFLP, and MSAP to analyze phenotypic, genetic, and epigenetic variation in natural populations of *V. negundo* var. *heterophylla* from an extensive variety of habitat environments. Two scoring approaches for MSAP marker data were conducted, and the results were consistent in most cases. The analysis demonstrated significant habitat‐related adaptation in phenotypic and genetic differentiation, suggesting an evident process of natural selection. However, we did not find a direct correlation between functional phenotypic and genetic variation, and Mantel tests and MMRR revealed a significant relationship between epigenetic and genetic or phenotypic variation. This result ultimately implied a potential intermediary role of epigenetic mechanisms in the adaption to heterogeneous habitats.

## CONFLICT OF INTEREST

None declared.

## AUTHOR CONTRIBUTION

LL, WG, and ND conceived the idea and designed the study. LL, CP, and ND contributed to the field survey. CP and LL performed the molecular laboratory work. LL, XG, and WG analyzed and interpreted the data. LL drafted the manuscript and all authors participated in manuscript modifications and gave final approval for publication.
